# Structure Tuning and Electrical Properties of Mixed PVDF and Nylon Nanofibers

**DOI:** 10.3390/ma14206096

**Published:** 2021-10-15

**Authors:** Petr Černohorský, Tatiana Pisarenko, Nikola Papež, Dinara Sobola, Ştefan Ţălu, Klára Částková, Jaroslav Kaštyl, Robert Macků, Pavel Škarvada, Petr Sedlák

**Affiliations:** 1 Department of Physics, Faculty of Electrical Engineering and Communication, Brno University of Technology, Technická 2848/8, 61600 Brno, Czech Republic; petr.cernohorsky94@gmail.com (P.Č.); Tatiana.Pisarenko@vut.cz (T.P.); papez@vut.cz (N.P.); sobola@vut.cz (D.S.); macku@vut.cz (R.M.); skarvada@vut.cz (P.Š.); sedlakp@vut.cz (P.S.); 2 Central European Institute of Technology, Purkyňova 656/123, 61200 Brno, Czech Republic; klara.castkova@ceitec.vutbr.cz (K.Č.); jaroslav.kastyl@ceitec.vutbr.cz (J.K.); 3 Department of Inorganic Chemistry and Chemical Ecology, Dagestan State University, St. M. Gadjieva 43-a, 367015 Makhachkala, Russia; 4 Directorate of Research, Development and Innovation Management (DMCDI), Technical University of Cluj-Napoca, Constantin Daicoviciu Street, No. 15, 400020 Cluj-Napoca, Cluj County, Romania; 5 Department of Ceramics and Polymers, Faculty of Mechanical Engineering, Brno University of Technology, Technická 2896/2, 61600 Brno, Czech Republic

**Keywords:** DSC, electrostatic spinning, FTIR, nanofibers, PA6, permittivity, PVDF, SEM, Raman spectroscopy, XPS

## Abstract

The paper specifies the electrostatic spinning process of specific polymeric materials, such as polyvinylidene fluoride (PVDF), polyamide-6 (PA6, Nylon-6) and their combination PVDF/PA6. By combining nanofibers from two different materials during the spinning process, new structures with different mechanical, chemical, and physical properties can be created. The materials and their combinations were subjected to several measurements: scanning electron microscopy (SEM) to capture topography; contact angle of the liquid wettability on the sample surface to observe hydrophobicity and hydrophilicity; crystallization events were determined by differential scanning calorimetry (DSC); X-ray photoelectron spectroscopy (XPS), Raman spectroscopy, and Fourier-transform infrared spectroscopy (FT-IR) to describe properties and their changes at the chemical level. Furthermore, for the electrical properties of the sample, the dielectric characteristics and the piezoelectric coefficient were measured. The advantage of the addition of co-polymers was to control the properties of PVDF samples and understand the reasons for the changed functionality. The innovation point of this work is the complex analysis of PVDF modification caused by mixing with nylon PA6. Here we emphasize that the application of nylon during the spin influences the properties and structure (polarization, crystallization) of PVDF.

## 1. Introduction

Polymeric material, which is lightweight, strong, and able to modify structure and properties, is particularly promising for producing large, flexible, low-power electronic devices. Fabricated functional nanostructures can generate electrical potential based on piezoelectric and triboelectric effect. In general, nanofiber power sources have become called nanogenerators. The invention of the nanogenerator presents the conversion of natural mechanical energies with irregular amplitudes and frequencies into electricity. One of the most effective methods for synthesising materials at the nanoscale is primarily electrostatic spinning. The ability to influence the content of crystalline phases and the associated dielectric and piezoelectric properties is crucial for specific material implementations and their practical use.

In recent years, piezoelectric nanogenerators (PENG) and triboelectric nanogenerators (TENG) have become increasingly attractive due to their sensitive working mechanism for energy conversion. The mechanism of PENG is based on piezoelectric polarization in materials induced by deformation, while TENG depends on electrostatic induction between two triboelectric layers [1,2,3]. Combining these two types of nanogenerators is therefore becoming desirable, and the demands for its improvement are growing.

### 1.1. Piezoelectric Nanogenerators (PENG)

There are rich sources of mechanical energy in the environment, which is the potential for electricity generation. It is now possible to convert low-intensity mechanical energy (e.g., human body movements) into electrical energy. For this purpose, new piezoelectric organic materials have been proposed to produce electricity from mechanical vibrations. A piezoelectric generator can obtain energy from ambient vibrations into usable electricity to power miniaturized devices that require a small amount of energy [1,4]. Compared to inorganic materials, only a small proportion of generators are based on polymeric materials, mainly due to poor piezoelectric coefficients [5].

Polymeric materials with a natural piezoelectric effect include mainly polyvinylidene fluoride (PVDF) and its trifluoroethylene copolymer (PVDF-TrFE) [1]. The piezoelectric effect is based on the hydrogen and fluorine atoms in vinylidene fluoride (VDF), which are perpendicular to the polymer backbone chain. The PDVF has three characteristic crystalline phases, namely α (alpha), β (beta), and γ (gamma). It is the α-phase that is usually formed in most polymerization situations. The content of the polar β-phase is essential information for the PVDF material because this phase shows the strongest piezoelectric character. To form the β-phase, the material must be electrically polarized by an electric field of the order of 100 MVs^−1^, or mechanically stretched. An increase in the crystalline β-phase can lead to a higher piezoelectric coefficient $d_{33}$. It can be noted that the copolymer of P(VDF-TrFE) with chemical formula $[-{\left({CH}_{2}-{CF}_{2}\right)}_{n}-{\left(CHF-{CF}_{2}\right)}_{m}-]$ more easily crystallizes into the β-phase due to steric factors. Electrostatic spinning has become a suitable method for the production of PVDF with a high β-phase content [4].

### 1.2. Triboelectric Nanogenerators (TENG)

Triboelectric generators combine two effects, contact triboelectrification and electrostatic induction. In the case of a triboelectric generator, the ability to generate triboelectrification and retain the generated charge on the dielectric surface is assessed. Although the charge transfer mechanism between two materials and the amount of charges transferred has not yet been known, the output voltage, current, and charges induced in a triboelectric generator can be measured [1].

Xue Pu et al. in their research in 2020 [6] included zinc oxide (ZnO) nanowires into electrospun PVDF and Nylon-11. The TENG construction was based on PVDF-ZnO/Nylon-11-ZnO materials. This study showed that ZnO nanowires were aligned along the fiber axis during electrospinning. Cooperative and mutual alignment of polymer chains with ZnO nanowires were achieved thanks to electrostatic spinning, which supports the formation of highly polar crystalline β-phase PVDF.

Polyamide 6 (PA6), also known as Nylon 6, is one of the most widely used polycrystalline polymers with a melting point of 223 ${}^{{}^{\circ}}C$. The material is often used due to its high chemical resistance, flexibility, high thermal stability, and low price. Amide and carbonyl groups are formed in PA6, which form the polar phases of the molecules. Because of them, hydrogen bonds are formed, which bind water, and cause hydrophilicity, greater mechanical strength, and resistance of PA6 nanofibrous structures [7].

By changing the content of the crystalline phase, the properties of PA6 can be modified. Mechanical and hydrophilic properties can be influenced by the increase in the content of crystalline phases. The structure of polyamide 6 can be significantly influenced by electrostatic spinning, where PA6 cools rapidly due to the stretching of the nanofiber. Thus, two characteristic crystalline structures of the α-phase and γ-phase are formed in the polymer, which cause the triboelectric properties of the material. The higher formation of the crystalline phase can also be supported by mixing PA6 with a clay material [6,8].

Nanofiber PA6 is still used for new applications in the field of electrical engineering (TENG), filtration, and textiles. It is also used as a component of solar and fuel cells [5,9].

## 2. Results and Discussion

Results in this experiment are based on the addition of nylon PA6 to polyvinylidene fluoride PVDF in the form of nanofibers and their investigation and comparison of these materials—PVDF, PVDF/PA6 mix, and PA6. After the successful production of three types of sheets of materials—PVDF, PVDF/PA6 mix, and PA6, they were all comprehensively analyzed. This part of the paper summarizes the set of results obtained by the methods listed in Section 3. The methods were chosen to complement each other. All successfully fabricated samples were included and compared in the measurement. Thus, many measurements were averaged for more accurate values from several materials, such as in Section 2.1, Section 2.2 or Section 2.7. Averaging was needed due to the fact that a produced sheet of fibers cannot always have homogeneously distributed material.

### 2.1. Topography of the Fabricated Material

Alignment of the nanofibers, their most common diameter, and defects was observed by electron microscopy. All of these properties affect different fiber behaviors, which can be prime to many applications. In Section 2.1.3, it has been found that the speed of rotation of the collector has the most significant effect on the alignment of the fibers. The electrospun PVDF material showed a higher amount of aligned fibers in one direction compared to PA6. Furthermore in Section 2.1.1, fiber diameters were evaluated from SEM images, where the PA6 material reached smaller diameters in the order of tens of nanometers, while the diameters of PVDF fibers were in the order of hundreds of nanometers. The mixed PVDF/PA6 material was more like the PA6 morphology in terms of fiber alignment, while the fiber diameters were similar in size to pure PA6 and PVDF materials and could be distinguished in the images. Fiber defects were observed for all materials in comparable amounts in Section 2.1.2. The most common defect was teardrop-shaped balls.

In the following text, these mentioned properties of fibers are examined in more detail and evaluated.

#### 2.1.1. Fiber Diameters

**Figure 1:**
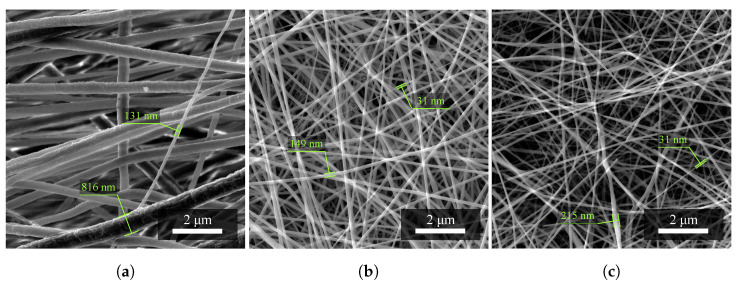
Investigation of the diameter of nanofibers of (**a**) PVDF material, (**b**) the combination of PVDF/PA6, and (**c**) PA6. Fibers of different sizes were randomly selected. Measurement parameters: acceleration voltage 5 kV, magnification 27.7 k×.

The value of the diameter of PVDF fibers (Figure 1a), which occurs in a given material structure, is most often around 350 nm with an upper limit of deviation (465 nm) and a lower limit of deviation (−219 nm). Fibers larger than the value of the upper limit of deviation, i.e., 815 nm, with given fabrication parameters can already be considered to be defects of the electrospinning process.

The value of the diameter of PVDF/PA6 fibers (Figure 1b), which occurs in a given structure of the mixed material, is most often around 176 nm with an upper limit of deviation (309 nm) and a lower limit of deviation (−136 nm). The SEM images show that the fibers of larger diameters belong to the PVDF material. On the contrary, the fibers of smaller diameters belong to PA6.

The value of the diameter of PA6 fibers (Figure 1c), which occurs in a given material structure, is most often around 62 nm with an upper limit of deviation (121 nm) and a lower limit of deviation (−43 nm). The fibers of this material have achieved better results in the electrospinning process in terms of smaller fiber diameters.

The entire measurement of all samples produced is then summarized in Table 1 below. As expected, it can then be seen that the average fiber diameter decreases with the addition of nylon.

**Figure 2:**
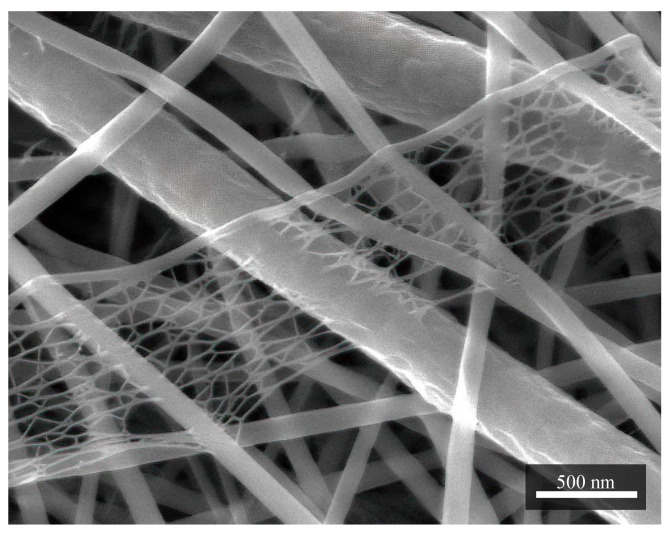
A structure of porous character resembling a cobweb. It is formed mainly with nylon. In this case, it is a sample in combination PVDF/PA6. Measurement parameters: acceleration voltage 5 kV, magnification 80 k×.

In Figure 2, it is also possible to observe structures of smaller dimensions around 8 nm, which are no longer fibrous. They are relatively porous, and the principle of their origin is not known yet.

#### 2.1.2. Fiber Defects

Defects in the electrospinning process can affect many parameters, such as [10]:

- low speed of the collector cylinder (different fiber thicknesses and their alignment),
- small distance between the emitter and the collector (the fibers begin to lose their spherical diameter and begin to stick together),
- or high voltage or high dose rate (formation of tear-shaped droplets).

There is a specific setting of spinning parameters for each material and its different concentrations in the precursor (as described in Section 3.1 and Section 3.2). In the experimental or laboratory spinning process, it is difficult to reduce all causes of undesirable defects, although the production parameters were chosen, emphasizing the quality and high β-phase of the fibers [11].

In the spun PVDF material, fibers with a larger diameter were mainly formed, which could be caused by a higher viscosity of the precursor or a small distance between the collector and the emitter—low collector speeds can be excluded in this case [12,13].

**Figure 3:**
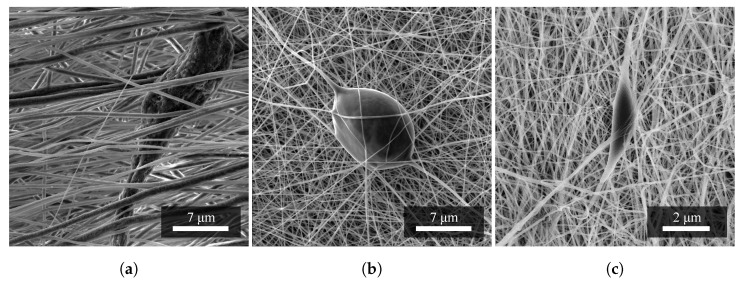
A set of images of nanofibrous materials based on (**a**) PVDF, (**b**) PVDF/PA6, and (**c**) PA6; and their typical occurring defects. It can be seen that the most common defect in the fibers is a tearshaped droplet in all materials. Measurement parameters: acceleration voltage 5 kV, magnification 9.4 k×.

Figure 3 shows an images of a defect of the so-called elongated droplet shape, which was caused by the properties of the precursor. Spun PA6 achieved an overall better fiber quality that was smooth and small in diameter. However, in PA6, elongated droplet defects can also be observed (Figure 3c). The mixed PVDF/PA6 nanofibers result from a combination of the properties mentioned above, and also contain teardrop defects (Figure 3b).

The occurrence of droplets is mainly influenced during fabrication by higher dosing, higher high voltage, or higher solution viscosity. As with PA6, these imperfections can occur in PVDF. However, any change in the parameter can also affect other properties of the fibers, so it is advisable to change them carefully. Nonetheless, the parameters used in this experiment are very promising, and efforts have been made to reduce the incidence of defects as much as possible.

#### 2.1.3. Fiber Alignments

**Figure 4:**
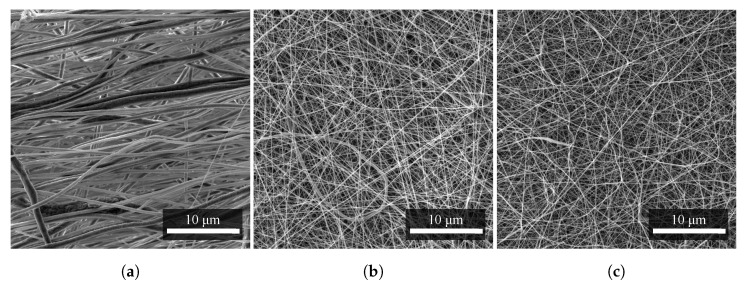
Comparison of density and alignment of nanofibers of (**a**) PVDF, (**b**) PVDF/PA6, and (**c**) PA6. All nanofibers were produced with the same technology at almost identical production parameters. Nevertheless, their difference can be seen at first glance, especially for PVDF. Measurement parameters: acceleration voltage 5 kV, magnification 5.5 k×.

From SEM images of the materials, the arrangement and density of the polymer fibers can be observed. Due to the smaller diameters, the PA6 fibers (Figure 4c) covered a particular area with a higher density, and the PVDF fibers (Figure 4a) were more aligned in a given direction due to the higher speed of the collector of the electrospinning apparatus [14,15]. Mixed PVDF/PA6 fibers (Figure 4b) are similar in morphology to PA6 in arrangement and surface coverage density [16].

### 2.2. Wettability of the Material

**Figure 5:**
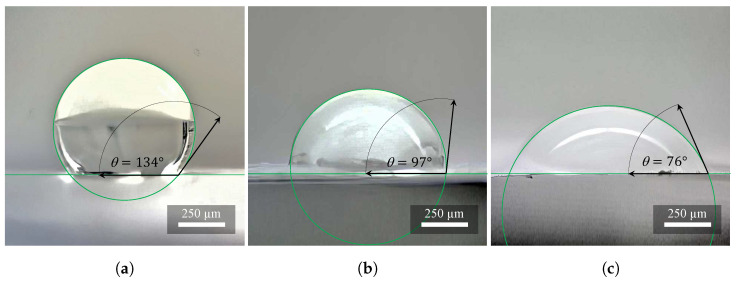
Investigation of hydrophobicity and hydrophilicity on (**a**) PVDF, (**b**) PVDF/PA6, and (**c**) PA6. The wettability of the samples increases with higher nylon concentration. These are average values from ten measurements.

According to the recorded data, the PVDF material (Figure 5a) can be categorized into hydrophobic materials. The contact angles of the individual drops at time t=4 s have the resulting averaged value θ=134${}^{{}^{\circ}}$, i.e., the value belonging to the hydrophobicity interval 90${}^{{}^{\circ}}$ to 150${}^{{}^{\circ}}$. After contact with the surface of the material at time t>600 s, the droplets almost did not change the contact angle, which means that the material did not adsorb the liquid.

Combinations of PVDF/PA6 materials (Figure 5b) can be categorized into hydrophilic materials. The contact angles measured of the individual drops at time t=4 s give the resulting average value θ=97${}^{{}^{\circ}}$, i.e., the value belonging to the hydrophobicity interval (90${}^{{}^{\circ}}$ to 150${}^{{}^{\circ}}$). The ratio of the percentage of PVDF or PA6 fibers in the PVDF/PA6 material determines whether the resulting material will be more hydrophobic or hydrophilic. After contact with the surface of the material, at time t≈45 s, the droplets adsorbed on the material about 50% of their volume, and then the rest of the volume was adsorbed more slowly. At time t>120 s, all droplets were adsorbed in total into the material (contact angle θ=0${}^{{}^{\circ}}$).

The PA6 material (Figure 5c) can be classified into hydrophilic materials according to our recorded data. The measured contact angles of the individual drops at time t=4 s have the resulting averaged value θ=76${}^{{}^{\circ}}$, i.e., the value belonging to the hydrophilicity interval (0${}^{{}^{\circ}}$ to 90${}^{{}^{\circ}}$). After contact with the surface of the material at time t≈10 s, the droplets adsorbed on the material about 50% of their volume, then the rest of the volume was adsorbed more slowly. At time t>30 s, all droplets were adsorbed in their entirety into the material (contact angle θ=0${}^{{}^{\circ}}$) [17].

### 2.3. Material Crystallinity Percentage Investigation

**Figure 6:**
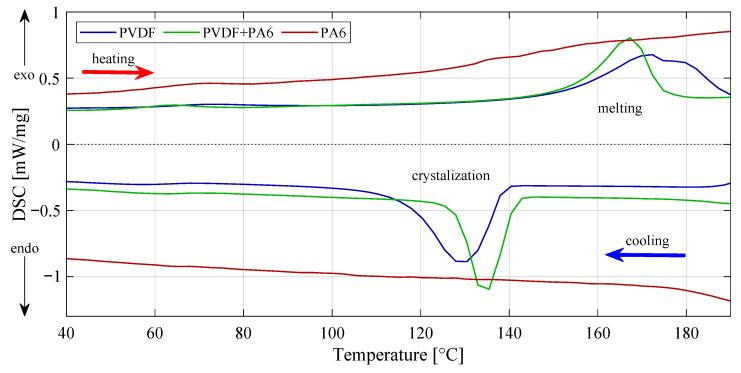
Exothermic and endothermic reactions can be observed from DSC curves. All three materials are presented here. Results are mainly used to calculate enthalpies of transitions by integrating the peak corresponding to a given transition. In this case, PVDF and PVDF/PA6 mixture only can be calculated.

Transition temperatures and enthalpies were determined using differential scanning calorimetry from peak integration in Figure 6. Crystalline phases $X_{C}$ were gained from Equation (1), where it is necessary to know heat of fusion of perfect crystalline PVDF $\Delta H_{f\varphi }^{*}=\;104.7\;J/g$ [18], and calculate enthalpy of fusion $\Delta H_{f}$, as was mentioned above [7,19]. Of course, PVDF fracture for the PVDF/PA6 was distinguished as first.

(1) $$X_{C}=\frac{\Delta H_{f}}{\Delta H_{f\varphi }^{*}}\;\cdot \;100\%$$

From the results, the enthalpy of pure PVDF reached 56.28 J/g with a final crystallinity of 53.75%. The mix of PVDF/PA6 reached an enthalpy of 39.37 J/g with a crystallinity of 60.16%. A sample of pure nylon was not included in the results.

### 2.4. Elemental Composition and Chemical State of the Material

**Figure 7:**
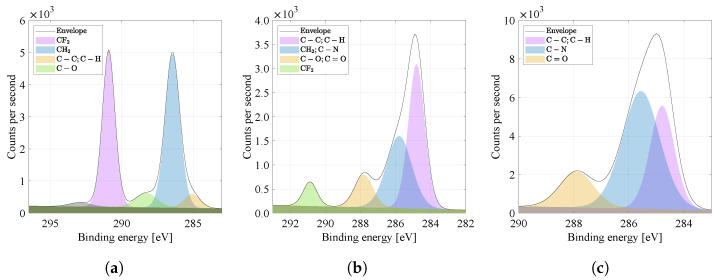
High energy resolution XPS spectra of C1s region. Nanofibers from the (**a**) PVDF, (**b**) PVDF/PA, (**c**) PA6 and their specific binding energies are compared.

By comparing the carbon spectra (binding energy of C1s) in Figure 7a–c, it is possible to observe more peaks in the range 284 to 294 eV, characteristic and near of carbon bonds, typical of PVDF and PA6 materials (see Table 2 [20]). For the PVDF material (Figure 7a), it is a $CF_{2}$ bond (290.9 eV), and for the PA6 material (Figure 7c), it is the dominant intensity of the chemical bond C−C (284.8 eV), which is the most represented in the PA6 monomer and is the basis (so-called chain backbone) of organic materials. Furthermore, the PA6 material is characterized by chemical bonds C−N (285.6 eV) and C=O (∼287.9 eV). The combination of the PVDF and PA6 nanofibers (Figure 7b) creates a material whose spectrum contains characteristic bonds of both materials [21]. The C−O bond can be attributed to the surface oxidation of the material.

**Figure 8:**
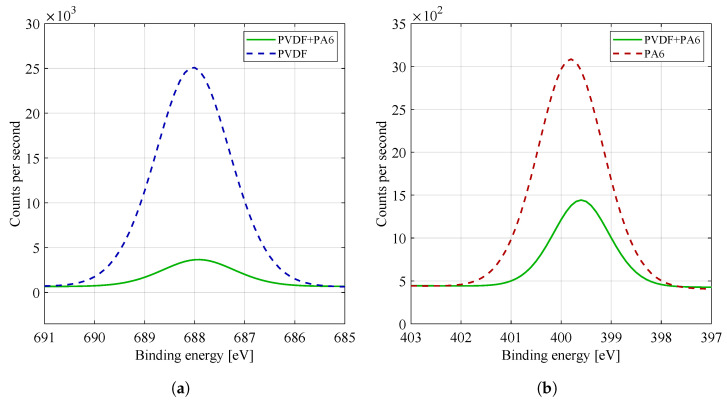
High energy resolution XPS spectra of (**a**) F1s and (**b**) N1s region. For practicality and a better overview, the curves of the given materials were plotted in one graph.

By comparing the spectra of the F1s high resolution binding energy in Figure 8a, i.e., the fluorine region, it is possible to see a dominant peak at 688 eV, characteristic for fluorine binding, typical for PVDF material (see Table 2). The spectra of F1s materials are mainly characterized by covalent and ionic bonds, with ionic bonds predominating. The PA6 material does not contain fluorine, so the F1s region has not been studied. For pure PVDF material, $CF_{2}$ binding C−F was detected at 688 eV. The same binding was recognized for the combined material PVDF/PA6 but in a lower concentration, as shown by comparing the intensities of the peaks of both materials. The intensity of the fluorine peak in the PVDF/PA6 composite material is dependent on the ratio of the given materials. Fluorine tends to cause significant chemical shifts in other elements, but within a given group of fluorine compounds (organic fluorine), the shifts in the F1s peak are negligible. The presence of fluorine atoms in polymers is the cause of its high temperature and chemical resistance (the chemical bond C−F is one of the strongest bonds in the macromolecule). The bond of fluorine and carbon also significantly affects hydrophobicity and hydrophilicity (in Section 2.2), where pure PVDF material (containing fluorine–carbon bond) showed hydrophobic properties.

By comparing the spectra of the N1s high resolution binding energy in Figure 8b, i.e., the nitrogen region, it is possible to see one dominant peak at 399.7 eV, characteristic for chemical nitrogen bonding, typical for PA6 material (see Table 2). The PVDF material does not contain nitrogen, so the N1s region was not investigated. For pure PA6 material, a chemical bond of C−N−H was detected at 400 eV. The same binding was recognized for the combined PVDF/PA6 material but at a lower concentration, as shown by comparing the intensities of the peaks of both materials. The intensity of the nitrogen peak in the mixed PVDF/PA6 material again depends on the ratio of the given materials.

**Figure 9:**
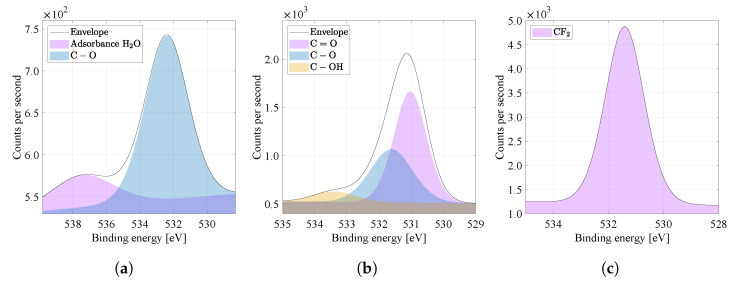
High energy resolution XPS spectra of O1s region. Nanofibers from the (**a**) PVDF, (**b**) PVDF/PA, (**c**) PA6 and their specific binding energies are compared.

By comparing the spectra of the O1s high resolution binding energy in Figure 9a–c, i.e., the oxygen region, it is possible to see more peaks in the range 529 to 540 eV, characteristic for oxygen bonds, typical for the PA6 material (see Table 2). The primary chemical structure of PVDF does not contain oxygen. For pure PA6 material, the C=O bond was detected at 531.4 eV. Atypical is the presence of a peak in the O1s region of pure PVDF around 530 eV and 534 eV. PVDF material can be attributed to surface oxidation and moisture, probably caused by contamination during sample handling under laboratory conditions. The combined material PVDF/PA6 is still dominated by the bond C=O ( 531 eV). Furthermore, C−O (531.6 eV) and C−OH (533.4 eV) bonds appear in PVDF/PA6, which can be attributed to the solvent molecules remain as contaminants or already mentioned surface oxidation and moisture.

### 2.5. Investigation of the Microstructural Formation of Material Molecules

**Figure 10:**
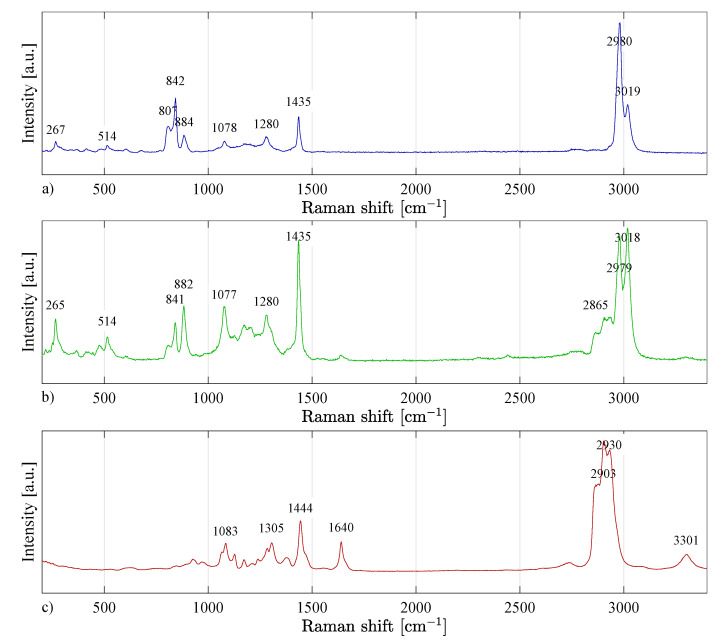
Raman spectra of (**a**) PVDF, (**b**) PVDF/PA6, and (**c**) PA6. In Figure (**b**) it is possible to successfully observe both PVDF and PA6 characteristic peaks.

The nanofibres based on the PVDF material contain the most crystalline β-phase, which confirms the electrostatic spinning theory that the process of electrostatic spinning of PVDF polymer promotes the formation of β-phase, thanks to which the fibers have piezoelectric properties [22,23]. The presence of the α and γ-phases were also confirmed and summarized in Table 3, and the spectrum recorded in Figure 10a.

By evaluating the Raman spectra of the PVDF/PA6 material (Figure 10b), the same intensities (peaks) of chemical bonds were identified as for the PVDF and PA6 materials. It follows that when PVDF and PA6 materials were mixed by electrospinning, the chemical bonds did not change significantly.

By evaluating the Raman spectra of the PA6 material (Figure 10c), individual chemical bonds in the molecular structure of the polymer were identified (Table 4). The particular crystalline phases of nylon cannot be precisely determined from the Raman spectrum-like PVDF. The content of γ-phase can be expected in the material [24].

### 2.6. Phase Fraction Determination from the Absorption Spectra

The determination of the individual phases of the PVDF material according to Equations (2)–(4) is based on the work by Cai et al. [25]. The authors summarize, evaluate, but also oppose the presentation of the results of PVDF analysis using FT-IR of many scientific papers dealing with the determination of α, β and γ-phase in PVDF. The typical peaks of α, β, γ-phases are listed in Table 5.

For the calculation of the percentage content of α, β and γ-phases for pure PVDF material, the following data were obtained from FT-IR:

- absorbance of β and γ-phases at 841 cm^−1^ ($A_{\beta \gamma }$),
- absorbance of α-phase at 763 cm^−1^ ($A_{\alpha }$),
- the difference between the absorbances of the corresponding peaks at 1275 cm^−1^ and the drop of 1260 cm^−1^ for the β-phase ($\Delta H_{\beta }$),
- the difference between the absorbances of the corresponding peaks at 1234 cm^−1^ and the drop of 1225 cm^−1^ for the γ-phase ($\Delta H_{\gamma }$).

The following applies to the relative fraction of the β and γ-phases:

(2) $$f_{\beta \gamma }=\frac{A_{\beta \gamma }}{K\;\cdot \;A_{\alpha }+A_{\beta \gamma }\;\cdot \;100},$$

where *K* is the ratio of the $A_{\beta \gamma }$ and $A_{\alpha }$ absorbencies.

The relative fraction of the α-phase is:

(3) $$f_{\alpha }=100-f_{\beta \gamma }.$$

The characteristic bands for the β and γ-phase often overlap (e.g., 840 cm^−1^ for β and 834 cm^−1^ for γ). The relative fraction of the β-phases ($f_{\beta }$) and γ-phases ($f_{\gamma }$) must therefore be further derived according to the following equations:

(4) $$f_{\beta }=f_{\beta \gamma }\frac{\Delta H_{\beta }}{\Delta H_{\beta }+\Delta H_{\gamma }},f_{\gamma }=f_{\beta \gamma }\frac{\Delta H_{\gamma }}{\Delta H_{\gamma }+\Delta H_{\beta }}.$$

**Figure 11:**
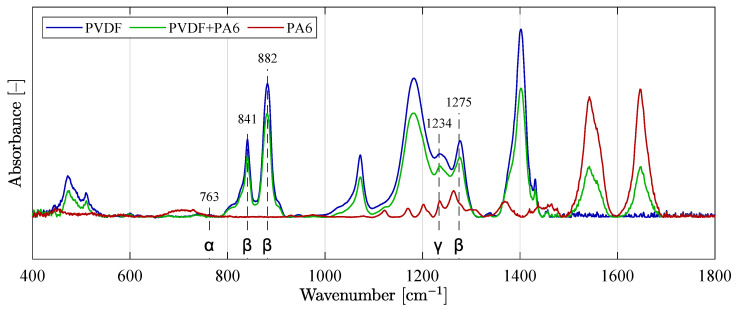
Absorption FT-IR spectra of three overlaid curves with typical and known phases.

The values for calculating the individual phases were obtained from the measured data of the individual spectra of the PVDF and PVDF/PA6 material. Figure 11 shows the curves of all three analyzed materials—PVDF, PVDF/PA6 mix, and PA6 for easier comparison of the content of individual phases α, β, and γ. Furthermore, some of the individual typical detected phases of the material are marked in the plot.

**Figure 12:**
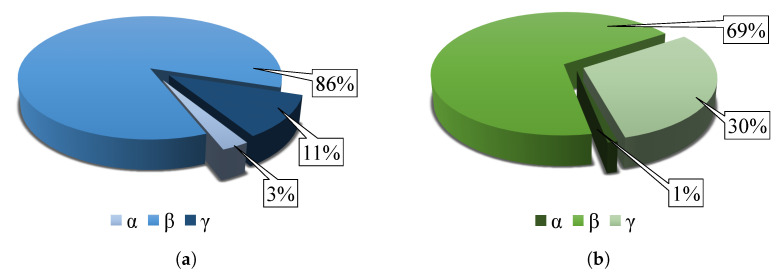
Comparison of calculated crystalline phase contents of (**a**) PVDF and (**b**) PVDF/PA6materials.

The results obtained by FT-IR analysis are summarized in Figure 12, which shows pie chart defining the contents of the individual α, β, and γ-phases in the materials of pure PVDF and the PVDF/PA6 mixture. The pure PVDF material has a phase fraction of α=2.87%, β=86.02%, and γ=11.2%. The mixed PVDF/PA6 has a phase fraction of α=1.06%, β=68.77%, and γ=30.17%.

The data show that the mixed β-phase of the PVDF/PA6 material is lower compared to pure PVDF. This is because the PVDF/PA6 mixture is composed of β-crystallizing PVDF and PA6 nanofibers, which do not crystallize in the β-phase, which reduces the β-phase content of the mixed nanofibers.

In contrast, the γ-phase occurs in higher amounts in the mixed PVDF/PA6 material than pure PVDF because the γ-phase in PVDF increases due to the addition of nylon. PA6 itself does not have a γ-phase. The arrangement of the PVDF also depends on the substrate or material with which the PVDF is mixed. The β-phase arises from the γ-phase under mechanical stress. In the case of a mixed sample, the PVDF fibers are less stretched and directed [26,27]. The content of α-phase can be neglected due to its amount in the samples.

### 2.7. Permittivity and Piezoelectric Constant d_33_

In the case of pure PVDF polymer and PVDF/PA6 mixture, the dielectric properties are influenced by the crystalline phase, fiber orientation, and temperature of the materials. The results of individual measurements differed by a negligible deviation, and the resulting values of repeated measurements were averaged for the given material.

Table 6 shows the resulting values of relative permittivity (real part of the permittivity) $\varepsilon^{\prime }$, dielectric loss factor (imaginary part of the permittivity) $\varepsilon^{\prime \prime }$, loss tangent tanδ, and capacitor capacity *C* with inserted dielectric at 1 and 100 kHz. According to the ISO table of reference value of relative permittivity of polymers [28], PVDF has a relative permittivity $\varepsilon^{\prime }=6–7.4$, and PA6 has a relative permittivity $\varepsilon^{\prime }=4–5$. A comparison of the reference values with the measured values shows that the nanofiber polymers have about 2.5× less relative permittivity than the reference values of the polymers. The reason for the decrease in relative permittivity can be attributed to the nanofiber structure, where the spaces between the fibers are filled with atmospheric air, which reference value of relative permittivity is close to the values of vacuum $\varepsilon^{\prime }=1.00054$ [29].

The measured values support the results of the previous FT-IR analysis (Section 2.6) that higher relative permittivity values are achieved by pure PVDF material compared to the PVDF/PA6 mixture and pure PA6, which may be due to the higher polar phase content of PVDF. PA6 nanofibers clearly reach the lowest relative permittivity values. From the results of relative permittivity (Table 6), it can be stated that the properties of all three materials are almost independent of the frequency of the applied voltage. In contrast, the dielectric loss factor and the loss tangent increased with increasing frequency [30].

All three crystalline phases together affect the resulting measured permittivity values. In addition to crystallinity, there are other effects on the dielectric constant, such as the presence of defects in the sample that tend to cause higher dipole momentum mobility and an increase in relative permittivity. Furthermore, the relative permittivity is strongly influenced by the presence of moisture ($H_{2}O$) in the material; with increasing humidity, the relative permittivity also increases [31].

**Figure 13:**
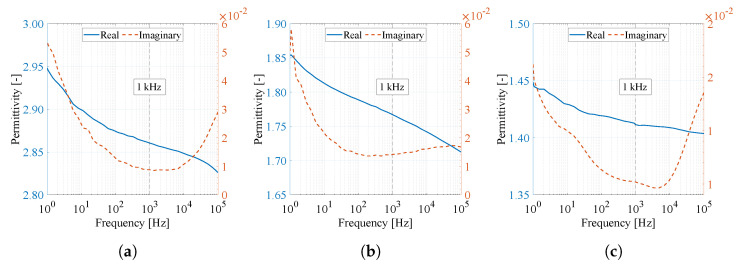
Real and imaginary permittivity curves of (**a**) PVDF, (**b**) PVDF/PA6, and (**c**) PA6 material as a function of frequency from 1 Hz to 100 kHz.

As an addition to Table 6, Figure 13 shows a complete overview of the permittivity of the real and imaginary parts as a function of frequency. From the characteristics, it is possible to observe a decreasing character of the curve with increasing frequency. Nevertheless, it can be argued that varying frequencies do not significantly affect changes in relative permittivity.

The piezoelectric constant $d_{33}$ measurements indicate that pure PVDF shows much higher values than its combination with PA6. This result was expected, and the amount of $d_{33}$ loss in the PVDF/PA6 mix was monitored, depending on the production parameters given in Section 3.1, and material parameters in Section 3.2. The average $d_{33}$ result for PVDF was 26.8 pC/N, and for PVDF/PA6 mix 8.2 pC/N, which is more than a threefold drop of $d_{33}$. This may also be affected by the β-phase loss described in Section 2.6.

## 3. Material and Methods

The experiment focused on the process of electrostatic spinning of two specific polymeric materials: PVDF and PA6, and their combinations. Furthermore, the prepared materials were subjected to analysis using the following methods: observation of materials by electron microscopy, determination of hydrophilicity and hydrophobicity of materials, differential scanning calorimetry, X-ray photoelectron spectroscopy, Raman spectroscopy, Fourier-transform infrared spectroscopy, determination of dielectric characteristics and piezoelectric coefficient $d_{33}$.

### 3.1. Methodology of NanoFiber Fabrication

The fibers were electrospuned using a 4SPIN machine (Contipro, the Czech Republic). The whole spinning process can be easily parameterized. Before that, however, it was necessary to assemble the needle (emitter) and rotating drum (collector) configuration. The setting of the parameters of the electrospinning process of the materials is recorded in Table 7 below.

The only different parameter during the electrospinning process seen in Table 7 was the choice of two needles for the PVDF/PA6 mix. The solutions of PVDF (based on DMSO) and PA6 (based on acids) are due to their solvents incompatible (polymer precipitation is initiated by the antagonistic solvent), and no conjoint solvent for the polymer exists. Thus, there was no option to prepare the composite from one mixed solution or via a co-axial needle.

As can be seen, the parameters in Table 7 also apply to the PVDF/PA6 mix. In this case, the same amount of solution comes out of both needles during electrospinning at one time, and their spinning time is the same. It can therefore be assumed that their distribution is half. This choice will allow a clear and balanced observation of the effects of both materials.

For each PVDF, PA6, and PVDF/PA6 mix, a carrier in the form of a conductive aluminum foil was used for easier material handling [32].

### 3.2. Materials and Their Parameters

The precursors were prepared by mixing the polymers with the solvents at 80 °C in a magnetic stirrer at 250 rpm, where their temperature and consistency were maintained. After proper preparation, the materials were immediately fabricated and measured.

A precursor containing a 20% PVDF polymer (Sigma Aldrich) with molecular weight of Mw=275.000 g/mol dissolved in DMSO/AC (dimethylsulfoxide/acetone) solvent in a 7:3 volume ratio was used for the piezoelectric nanofibers. For triboelectric nanofibers, a precursor containing a 20% PA6 polymer (Alfa Chemicals) with molecular weight of Mw=35.000 g/mol dissolved in a FA/AA (formic acid/acetic acid) solvent in a 4:1 volume ratio was used.

Dimethylsulfoxide (DMSO) solvent was chosen due to its higher polarity, which tends to enhance the polarization of fibers during the electrospinning process. The volatility of the solvent was sufficiently increased by acetone adding.

The used molecular weights, as well as solvent mixtures, were properly chosen and experimented in order to prepare spinnable precursors forming as much as possible defect-free fibers of sub-micrometer width. Our previous unpublished experiments showed the 20% solution of PVDF 275 and the 20% solution of PA6 are optimal for the intended study of PVDF/PA6 composites.

For the preparation of combined materials (so-called PVDF/PA6 mix), the same precursor concentrations were used as mentioned above. A solid cylindrical metal collector and two static needles for each precursor were used to configure the electrospinning apparatus.

### 3.3. Methods Used to Investigate the Formed Nanofibers

The following eight methods were used for a comprehensive examination of all fabricated materials. Each instrument was selected so that the results from every method could complement each other and confirm the resulting findings as outlined at the beginning of Section 2.

#### 3.3.1. Scanning Electron Microscopy (SEM)

The fiber structure was observed by scanning electron microscopy on a LYRA3 microscope (Tescan, Brno, the Czech Republic) and a Helios NanoLab 660 (Thermo Fisher Scientific, Waltham, MA, USA). Due to the polymeric nature of the sample, the fibers of the material begin to charge and repel with each other as the charge accumulates, resulting in the movement of the fibers making it difficult to focus and scan at high magnification. Therefore, the samples for analysis were carbonized on a Coater EM ACE600 instrument (Leica Microsystems, Wetzlar, Germany) for analysis. Several images were selected for each sample, and the mean of ten randomly selected fibers was measured. Newly occurring defects were also observed. The following parameters were chosen for all SEM observations: detector SE, acceleration voltage 5kV, working distance 9 mm (LYRA3) and 4 mm (Helios), magnification from 5 k× to 80 k×.

#### 3.3.2. Hydrophobicity and Hydrophilicity

The measurement aimed to compare the three analyzed materials (PVDF, PA6, and the combination of PVDF/PA6) with respect to their liquid watertightness, and subsequent categorization. It is known that the contact angle θ measured on adsorbent materials decreases with increasing droplet adsorption. The influence of three parameters related to adsorption (adsorption rate, droplet size, and residence time) on the measured contact angle is comprehensively captured by the relative volume of the adsorbed droplet, i.e., the percentage of the droplet volume penetrated the material. The watertightness of a 3 μL droplet on PVDF, PA6, and PVDF/PA6 combinations with very different liquid adsorption rates was analyzed.

The test liquid was distilled water. The evaporation effect of the liquid used was negligible due to the measurement time and was therefore not included in the evaluation. The measurement was performed using a See System E (Advex Instruments, Brno, the Czech Republic) instrument with subsequent evaluation of the contact angle from the images using the See Software 7.0. Ten drops ( 3 μL) were gradually applied to the given test material using a dosing micropipette. Then, for ten drops of the three tested materials, the contact angle was measured at time t=4 s. Means that the time from the contact of the drop with the surface of the material was measured. To determine the contact angle from the images, the methodology was used using three manually determined points; thus a circle was created, which copies the contour of the drop shape at the liquid-gas interface with the highest possible accuracy.

#### 3.3.3. Differential Scanning Calorimetry (DSC)

Using this thermoanalytical technique, it was possible to detect phase transitions such as melting or crystallization. The crystallinity percentage of the observed specimens was calculated and accurately determined for each type of material produced. The DSC 204 F1 (NETZSCH, Germany) was chosen for the measurement. The experiment was carried out in the temperature range of 25 to 200 °C with a heating rate of 10 °C/min and under an argon flux of 20 mL/min.

#### 3.3.4. X-ray Photoelectron Spectroscopy (XPS)

The XPS spectra of the materials were acquired with the AXIS Supra X-ray photoelectron spectrometer (Kratos Analytical, Manchester, UK). The emission current was set to 15 mA. All the values of the recorded graphs were calibrated according to the C–C binding reference value (284.8 eV). The plotted spectra already represent a fitting of the measured data [33].

#### 3.3.5. Raman Spectroscopy

The properties of the polymer fibers were studied using a WITec Alpha 300R (WITec, Ulm, Germany) [34]. By analyzing the Raman spectrum, it was possible to identify the crystalline phases present in the sample. Raman spectroscopy provides additional information to FT-IR and XPS analyzes.

By evaluating the Raman spectra of the studied material, the individual crystalline phases of the α, β and γ polymer were identified. The excitation wavelength of the instrument was set to 532 nm (green laser) and the laser power was 6 mW, while the number of accumulations was 30× at an integration time of 7 s using a lens with a magnification of 100×.

#### 3.3.6. Fourier-Transform Infrared Spectroscopy (FT-IR)

Qualitative and quantitative information of the crystal structure of the analyzed materials was obtained using Fourier-transform infrared spectroscopy. Based on the measured spectra and specific values of the peaks, it is possible to determine the percentage of crystalline β-phase (especially in the case of PVDF material), the presence of which causes a piezoelectric effect. The piezoelectric properties can also be partly caused by the γ-phase.

The spectrum was measured on a Vacuum FT-IR Vertex70v spectrometer (Bruker, Billerica, MA, USA), and the values of the intensity of the absorbed energy depending on the wavelength of the infrared radiation were recorded in the spectra graphs. The absorbance spectrum was measured with a wavelength resolution of 0.5 cm^−1^. Background noise was subtracted from the individual curves for more accurate analysis data.

There is a characteristic wavelength band for each of the α, β, and γ-phases of PVDF (Table 5). The spectra of the PA6 materials and the PVDF/PA6 mix were measured for comparison with the pure PVDF material. The PA6 material crystallizes only in the α and γ-phases, so it does not form the β-phase.

#### 3.3.7. Dielectric Permittivity Measurement

Dielectric measurements were performed with the Novocontrol Alpha analyzer (Novocontrol Technologies, Montabaur, Germany) and the Agilent 16451B electrode system (Agilent Technologies, Santa Clara, CA, USA) using an AC voltage of 100 mV in the frequency range 1 Hz to 100 Hz at room temperature to obtain basic information on dielectric properties and, in particular, the relative permittivity of the material $\varepsilon^{\prime }$.

The measured dielectric properties of the samples help to elucidate the overall behavior of the electrical properties. The measured materials reached a thickness of about 40 μm. The dielectric properties were repeatedly measured (10×) for each material, each at a different location.

#### 3.3.8. Piezoelectric Coefficient Measurement

How material converts mechanical stress into electrical charge was described by piezoelectric constant $d_{33}$, which was measured by quasi-static piezo $d_{33}$-meter, model ZJ-3B (HC Materials Corporation, Bolingbrook, IL USA) in the range of 1 to 200 pC/N with resolution 0.1 pC/N and 2% relative error. To further reduce the measurement error, a minimum of ten pieces of sample from different parts of the spun sheet of fibers were selected for each material, and their value was averaged. Pure PVDF and PVDF/PA6 mix which may exhibit a piezoelectric effect, were measured. An ideal contact was made between the $d_{33}$-meter jaws and the sample, i.e., such that the applied mechanical force did not perforate the sample and also such that there was no triboelectric effect that could distort the measurement.

## 4. Conclusions

This experiment aimed to compare the parameters and properties analyzed of the polymeric fibrous nanomaterials PVDF, PA6, and their combination PVDF/PA6.

For produced fibers, average thickness decreased after the addition of PA6. On the contrary, their density increased. The electron microscope also detected fiber defects occurring in all three materials. These were often droplets in the fibers, which are affected by different production parameters or even the humidity of the environment.

The materials were also compared in terms of hydrophobicity and hydrophilicity, where pure PVDF showed strong hydrophobic to almost superhydrophobic properties, while PA6 achieved excellent hydrophilic results. Combining these two polymers resulted in PVDF/PA6 mix with properties at the boundary between hydrophobicity and hydrophilicity.

By evaluating FT-IR, XPS and Raman spectra, crystalline phases (α, β, and γ), and chemical bonds in material molecules were identified. The most crystalline β-phase contained pure PVDF material parse-numbers=false β=86.02%, while the mixed PVDF/PA6 material contained more phase parse-numbers=false γ=30.17%. The α-phase occurred in the given materials in a negligible amount, about 1% to 2%. The results confirm the theory that the process of electrostatic spinning of polymers supports the formation of crystalline phases, thanks to which the material has piezoelectric and triboelectric properties. In the spectra of the pure PA6 material, it was difficult to recognize individual crystalline phases; therefore, the vibration levels of individual chemical bonds of nylon polymer were evaluated. The XPS method was the most accurate method for determining chemical bonds in materials.

From the measurement of dielectric properties, it is evident that the nanofibrous structure of materials reduces the relative permittivity. Pure PDVF had about two times higher relative permittivity than mixed PVDF/PA6 material. The presence of atmospheric air between the fibers of the material also contributed to reducing the relative permittivity of the material. The loss of the $d_{33}$ coefficient on the PVDF/PA6 mix was also confirmed. Its value was up to three times less than pure PVDF.

Variation of PVDF and PA6 allows the material to obtain very demanded structural (phase composition, crystallinity) and functional (mechanical, electrical) properties. On the other hand, mixing reduced the relative permittivity and probably decreased the β-phase content. However, the material still retained an overall high content of β and γ-phase, making it suitable for triboelectric and piezoelectric applications. The material needs to be further optimized and studied for future practical applications. The described results may bring new possibilities to the use of mixed PVDF/PA6 as an easily modifiable, inexpensive and accessible material for textile applications and nanogenerators.

## Figures and Tables

**Table 1:** Evaluation of measured data sets of nanoFiber diameters.

Material	Minimum [nm]	Maximum [nm]	Mean [nm]
PVDF	131	816	350
PVDF/PA6	40	485	176
PA6	19	123	62

**Table 2:** Reference values of carbon binding energies [20].

Element	Chemical Bond	Binding Energy [eV]
Carbon [C]	C−C	284.8
	C−O	∼286
	C=C	∼289
	$CF_{2}$	∼292
	$CF_{3}$	∼294
Nitrogen [N]	$C-NH_{2}$	∼400
Oxygen [O]	C−O	531.5–532
	C=O	∼533
Fluorine [F]	Organic fluorine	688–689

**Table 3:** Identification of bonds of crystalline phases of PVDF.

Molecular Structure	Raman Shift [${\mathrm{cm}}^{-1}$]
α-phase	807, 884, 1435
β-phase	842, 884, 1078, 1280, 1435
γ-phase	842, 884, 1435

**Table 4:** Identified and presumed chemical bonds in PA6 material.

Raman Shift [${\mathrm{cm}}^{-1}$]	Bond Formations
926	C−CO stretching
1062	C−C stretching
1083	C−C stretching
1127	C−C stretching
1285	C−N stretching, N−H bending (Amid III)
1305	$CH_{2}$ rotation
1444	$CH_{2}$ bending
1467	C−N stretching, N−H bending (Amid II)
1640	C=O stretching

**Table 5:** Characteristic peaks of α, β, γ-phases for PVDF.

Phase	Wavenumber [${\mathrm{cm}}^{-1}$]
α	410, 489, 532, 614, 763, 795, 854, 975, 1149, 1209, 1383, 1423
β	445, 473, 510, 841, 1275, 1432
γ	431, 482, 811, 1234, 1429

**Table 6:** Dielectric properties of materials at 1 and 100 kHz.

Material	*f* [kHz]	*C* [pF]	tanδ [–]	$\varepsilon^{\prime }$ [–]	$\varepsilon^{\prime \prime }$ [–]
PVDF	100	12.7	0.00949	2.8259	0.020572
	1	12.9	0.00281	2.8604	0.008770
PVDF/PA6	100	7.74	0.01125	1.7125	0.016724
	1	8.01	0.00922	1.7669	0.014196
PA6	100	1.21	0.01184	1.4037	0.013556
	1	1.22	0.00367	1.4114	0.005190

**Table 7:** Set parameters for spinning production of PVDF, PVDF/PA6, and PA6 on the 4SPIn device.

Parameter	Value	Unit
Emitter type	one needle (two needles for PVDF/PA6)	–
Needle size	1.4 (G17)	mm
Syringe volume	10	mL
Precursor dosing	15	μL/min
Collector type	rotating drum	–
Collector speed	2000	rpm
Distance (emitter-collector)	15	cm
High voltage	50	kV
Humidity (in the chamber)	20–26	%
Temperature (in the chamber)	22–25	${}^{{}^{\circ}}C$
Air flow rate	–	L/min
Heater	–	${}^{{}^{\circ}}C$
Process time	90	min

## Data Availability

Samples of PVDF, PVDF/PA6 and PA6 nanofibers are available on demand from Nikola Papež. E-mail: papez@vut.cz.

## Abbreviations

The following abbreviations are used in this manuscript:

AA	Acetic acid
AC	Acetone
DMSO	Dimethylsulphoxide
DSC	Differential scanning calorimetry
FA	Formic acid
FT-IR	Fourier-transform infrared spectroscopy
PA6	Polyamide-6
PENG	Piezoelectric nanogenerators
PVDF	Polyvinylidene fluoride
SE	Secondary electron
SEM	Scanning electron microscopy
TENG	Triboelectric nanogenerator
XPS	X-ray photoelectron spectroscopy
